# Circulating microparticles are associated with plaque burden and cause eNOS uncoupling in patients with carotid atherosclerosis

**DOI:** 10.3389/fphar.2022.976644

**Published:** 2022-11-03

**Authors:** Xiaowan Han, Tong Li, Tieshan Wang, Baofu Wang, Yang Li, Lei Wang, Ziwen Lu, Aiming Wu, Lisong Liu, Guozhong Pan, Mingjing Zhao

**Affiliations:** ^1^ Dongzhimen Hospital, Beijing University of Chinese Medicine, Beijing, China; ^2^ Beijing Research Institute of Chinese Medicine, Beijing University of Chinese Medicine, Beijing, China

**Keywords:** carotid atherosclerosis, circulating microparticles, endothelial dysfunction, endothelial nitric oxide synthase uncoupling, leukocyte-derived microparticle

## Abstract

**Aims:** The study aimed to evaluate the correlation of different microparticle (MP) phenotypes with plaque burden and their diagnostic value and preliminarily explore the role of MPs in atherosclerosis (AS).

**Methods:** Carotid intima-media thickness (CIMT) and maximal plaque area in 23 patients with carotid atherosclerosis (CAS) and 22 healthy subjects were measured by ultrasound. Transmission electron microscopy, nanoparticle tracking analysis and western blot were used to identify MPs. Flow cytometry assay measured absolute number of MPs, and receiver operating characteristic (ROC) analysis was used to assess the relationship between plaque burden and MPs. To study the preliminary mechanism of MPs in AS, MPs were administered to 32 male Kunming mice, which were randomly divided into control, CAS, healthy, and tetrahydrobiopterin (BH4) groups. Hematoxylin-eosin staining, immunohistochemistry staining, and Western blot were adopted to detect relevant indexes 24 h after the injection.

**Results:** The plasma levels of CD45^+^ leukocyte-derived microparticle (LMP), CD11a^+^ LMP, CD11a^+^/CD45^+^ LMP, and CD31^+^/CD42b^+^ platelet-derived microparticle (PMP) in CAS patients were significantly higher than those in healthy subjects, and were positively correlated with the maximal plaque area. Moreover, the levels of CD11a^+^ LMP, CD11a^+^/CD45^+^ LMP were also positively correlated with CIMT. The area under the ROC curve of the four MPs was 0.689, 0.747, 0.741, and 0.701, respectively. Compared with healthy subjects, MPs from CAS patients resulted in a significantly lower expression of endothelial nitric oxide synthase (eNOS) dimer/monomer, and BH4 could improve eNOS uncoupling. Moreover, the level of VCAM-1 in intima in the CAS group was significantly higher than in the other three groups.

**Conclusion:** CD11a^+^ LMP and CD11a^+^/CD45^+^ LMP might be potential biomarkers for CAS prediction. BH4-related eNOS uncoupling occurs in CAS patients, and circulating MPs from them lead to endothelial dysfunction through eNOS uncoupling.

## Introduction

Atherosclerosis (AS), the underlying pathology of cardiovascular diseases, is characterized by atherosclerotic and fibrous plaques. It is caused by the interaction between endothelial dysfunction, lipid metabolism disorder, and inflammatory cell infiltration (Yahagi et al., 2016; Basatemur et al., 2019). Nowadays, carotid ultrasound is the main method to diagnose AS, but it cannot sufficiently predict prognosis in the early stage.

In this nano era, nanoparticles, nano spindles (He et al., 2022), nanofibers (Yu et al., 2022), and microparticles (MPs) are hot and popular topic for drug delivery. Among them, MPs, small-membrane vesicles (diameter: 100–1000 nm), that consist of oxidized phospholipids and cell-specific proteins (Chen et al., 2020), have attracted increasing attention (Boulanger et al., 2017). MPs are involved in many pathological processes of AS. Some reports have demonstrated that circulating MPs level is a novel biomarker of endothelial dysfunction, reflecting clinical outcomes in several cardiometabolic diseases (Fu et al., 2015; Lin et al., 2017). MPs may promote AS development and progression by impairing endothelium-dependent vasodilatation and accelerating inflammatory cascade and atherosclerotic lesion formation (Ye et al., 2017), which further increase the level of circulating MPs in patients, forming a vicious circle.

Endothelial dysfunction is an initial step in early AS and also involved in subsequent lesion formation, plaque progression, and occurrence of atherosclerotic complications (Flammer et al., 2012). Nitric oxide (NO) is synthesized by L-arginine and oxygen molecules under the action of nitric oxide synthase. When endothelial nitric oxide synthase (eNOS) is uncoupled, it results in impaired bioavailability of NO, which aggravates endothelial dysfunction (Margaritis et al., 2013). Tetrahydrobiopterin (BH4) is an important cofactor of eNOS, promoting the dimerization of eNOS and restoring eNOS uncoupling (Sugiyama et al., 2009; Li et al., 2015). In the absence of BH4, eNOS cannot catalyze the oxidation of L-arginine to L-citrulline and NO leading to endothelial dysfunction (Vásquez-Vivar et al., 2003).

Can circulating MPs be used as a favorable indicator to assess the trend of plaque burden during atherosclerotic plaque formation? To answer this question, this study aimed to evaluate the relationship between the level of MPs and carotid plaque burden, assess the diagnostic value of MPs and find new biomarkers in AS patients. At the same time, the effects of circulating MPs on eNOS were preliminarily investigated.

## Materials and methods

### Patients

Twenty-three patients with carotid atherosclerosis (CAS) (excluding those with diabetes, cancer, acute inflammation, pregnancy, and liver and kidney dysfunction) and 22 healthy subjects were enrolled, and their baseline characteristics were recorded. CAS included unilateral or bilateral carotid intima-media thickness (CIMT) of ≥0.9 mm or carotid plaque formation with local CIMT increased to 1.3 mm but not significant stenosis (≤50%) (Hu., 2008). The maximal plaque area was defined as the area of the largest plaque in the left and right common carotid arteries and the sinus. Healthy subjects were included through a health examination without carotid plaque, hypertension, hyperlipidemia, and diabetes. All subjects were from the Physical Examination Center of Dongzhimen Hospital, Beijing University of Chinese Medicine. The study received consent from the subjects and passed the review by the ethics committee of Dongzhimen Hospital, Beijing University of Chinese Medicine (ethical number: DZMEC-KY-2017-80).

### Blood sampling and biochemical analysis

Venous blood (6 ml) samples were collected in the morning after overnight fasting and were injected into serum and trisodium citrate glass tubes for serological index detection, flow detection, and mice experiments. Platelet-poor plasma was obtained within 4 h by two serial centrifugations (300 g, 20 min, 16°C and 1,500 g, 20 min, 4°C), and 50 μl of it was used for flow detection. MPs were isolated by ultra-centrifuging at 21,000 g for 120 min at 4°C of the remaining part, re-suspended with saline, and stored at 4°C. MPs were injected into the tail vein of mice to observe the direct impact on the circulatory system. Blood lipid levels were measured by routine biochemical methods in the physical examination center of Dongzhimen Hospital, and the level of eNOS and BH4 was detected by enzyme-linked immunoassay (ELISA).

### Carotid ultrasound measurement

The subjects were examined in the supine position with the head tilted to the side opposite to the one being scanned. The physician placed the ultrasound probe on the corresponding body surface of the test vessel and performed a continuous scan from the proximal part to the distal part (HITACHI 6500). The scope of ultrasound examination included the bilateral common carotid artery, carotid bifurcation, and internal carotid artery (Min et al., 2004).

### Microparticles characterization

MPs were extracted as described above, and identified by transmission electron microscopy. A 5 μl sample was added to copper grids and incubated for 5 min. Then a drop of 2% uranyl acetate was added to the copper grids and incubated for 1 min. After drying for 20 min, grids were imaged with 80 kV transmission electron microscope (Tecnai G2 Spirit BioTwin, FEI, United States). Nanoparticle tracking analysis (qNano, IZON Science, New Zealand) was used to measure the size distribution of MPs. Western blot was used to examine MPs-associated protein markers Annexin V. The sample of MPs was lysed on ice for 10 min and then centrifuged to collect supernatant. The protein content in MPs was measured with BCA Protein Assay Kit.

### Flow cytometry

The platelet-poor plasma (50 μl, reserved in the above-described procedure) was incubated with 50 μl FC-block-staining buffer (BD-564219) for 10 min at room temperature. Then, the above-mentioned mixture was incubated with conjugated monoclonal antibodies CD31-PE (BD-555446), CD42b-FITC (BD-555472), CD11a-APC (BD-550852), and CD45-APC-Cy7 (BioLegend-368516) for 20 min in the same environment. After antibodies labeling, the mixture was diluted in 200 μl phosphate-buffered saline and then added to the Trucount™ Absolute Counting Tubes (BD-340334) to determine the absolute MP number. The MPs detection was performed on a flow cytometer (FACSCanto II, BD), and protocol standardization was based on a blend of fluorescent size-calibrated beads 1 μm (Sigma-L2778). Leukocyte-derived MPs (LMPs) were defined as CD45^+^, CD11a^+^, and CD11a^+^/CD45^+^ (Anne, 2012; Schwarz et al., 2018) while platelet-derived MPs (PMPs) and endothelial cell-derived MPs (EMPs) were defined as CD31^+^/CD42b^+^ and CD31^+^/CD42b^−^ (Amabile et al., 2012; Camaioni et al., 2013), respectively. The results were expressed as the number of MP/μl of plasma.

### Animals and intervention

A total of 32 male Kunming mice (20–22 g) were purchased from Charles River Laboratory Animal Technology Co. Ltd. (Beijing, China) for preliminary mechanism research. They were randomly divided into control group (saline), CAS patient MPs group, healthy subject MPs group, and BH4 (69056-38-8, HARVEYBIO, Beijing, China) group (3 days gavage, 10 mg/kg). The MPs injection method was performed as follows. MPs were injected through the tail vein of mice with a 1 ml syringe (Sheu et al., 2015). After 24 h, the blood was drawn by removing the eyeballs, and the tissues of the heart, thoracic aorta, and abdominal aorta were taken immediately. The animal research was reviewed and approved by the Animal Care and Welfare Committee of Dongzhimen Hospital, Beijing University of Chinese Medicine (ethical number: 17–35).

### Histology

Hematoxylin and eosin (HE) staining was performed as previously reported (Chang et al., 2015). 3 μm-thick paraffin aorta sections were stained with HE and then photographed at ×100 and ×400 magnification with a microscope for morphological analysis including intimal integrity and structure of media and adventitia.

### Immunohistochemistry assay

All samples were repaired in a microwave and incubated in 0.3% hydrogen peroxide before using primary [vascular cell adhesion molecule-1 (VCAM-1), BA3840, Boster, China] and secondary antibodies (Zhongshan Golden Bridge Biotechnology Company, China). Positive reactions were visualized by incubating the slides with diaminobenzidine as a substrate in the cytomembrane. Images were photographed with SPOT V3.0II software and the average optical density was measured and analyzed by Image-Pro Plus 6.0.

### Western blot analysis

The purity and concentration of circulating tissue protein were determined before electrophoretically transferred the mixture onto a polyvinylidene difluoride (PVDF) membrane. Nonspecific binding was blocked by the incubation in a blocking buffer [5% nonfat milk in TBST (TBS containing 0.05% Tween 20)]. The membranes were incubated with the indicated primary antibodies (anti-mouse eNOS antibody; diluted 1:1000, ab76198, Abcam, Cambridge, United Kingdom) overnight at 4°C. HRP-conjugated anti-rabbit immunoglobulin (Ig)G was used as a secondary antibody (diluted 1:5000, BA1050, Boster Biological Technology, Wuhan, China) for 1 h at room temperature. Bands were visualized by chemiluminescence, and the image analysis software ImageJ was used to quantitatively analyze the gray value of the target protein and background.

### Statistical analysis

Data were expressed as median (25th–75th percentile) or mean ± standard deviation (SD) and analyzed using SPSS, version 20.0. Student’s *t* tests or Mann-Whitney *U*-test was used for normally or nonnormally distributed continuous variables comparison. Bivariate correlation analyses were performed by using Spearman rank correlation test, and the accuracy of diagnostic tests used the area under the receiver operating characteristic (ROC) curve (AUC, 95% confidence interval). A value close to 0.5 indicated the test failed, while 1 indicated an almost perfect result. The cut-off value for the number of MPs was determined using Youden’s test. A value of *p* < 0.05 was considered statistically significant.

## Results

### Baseline characteristics of subjects

Table 1 shows the demographic and clinical characteristics of subjects, including age, sex, complications (hypertension and hyperlipidemia) and ultrasound findings (CIMT and maximal plaque area). The age of healthy subjects was 50 (47.5–59.5), and that of CAS patients was 62 (61–65.5), with a significant difference between the two groups (*p* < 0.01). There were 20 females and two males in the healthy group, and 20 females and three males in the CAS group, without a significant difference between the two groups (*p* > 0.05). Of 23 subjects in the CAS group, 17 had hyperlipidemia, and 17 had hypertension. The CIMT of CAS patients was 0.12 (0.11–0.13) cm, while that of healthy subjects was <0.09 cm. The maximal plaque area of CAS patients was 0.14 (0.07–0.24) cm^2^.

**TABLE 1:** Baseline characteristics of subjects.

Characteristics	CAS	Healthy	*p* value
Age	62 [61–65.5]	50 [47.5–59.5]	0.000
Gender (female/male)	20/3	20/2	1.000
Hyperlipidemia	17 (23)	0 (22)	—
Hypertension	17 (23)	0 (22)	—
CIMT (cm)	0.12 [0.11–0.13]	<0.09	—
Maximal plaque area (cm^2^)	0.14 [0.07–0.24]	—	—

Measurement data were expressed as the median [25th–5th percentile].

*p* < 0.05 was considered statistically significant.

### Identification of microparticles

**FIGURE 1:**
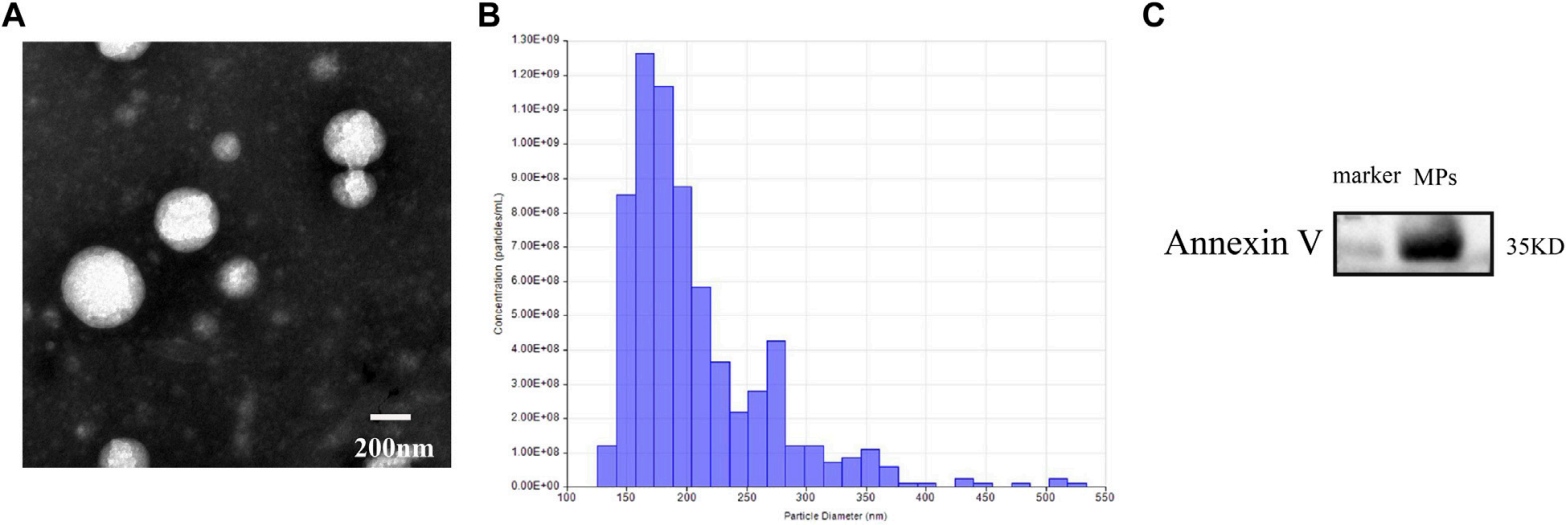
**(A)** transmission electron microscopic images of MPs. **(B)** nanoparticle tracking analysis showing the particle distribution of different size of MPs. **(C)** western blot analysis showing the biomarker of MPs.

Transmission electron microscopic images showed that most of MPs had a spherical morphology. Nanoparticle tracking analysis confirmed that the isolated MPs had a diameter primarily distributed between 126 and 519 nm. The vesicles contained the MPs-associated protein marker Annexin V. These results indicated that the isolated vesicles released from venous blood were MPs (Figure 1).

### The quantitative expression of leukocyte-derived microparticle, platelet-derived microparticle, and endothelial cell-derived microparticle in carotid atherosclerosis patients and healthy subjects

**FIGURE 2:**
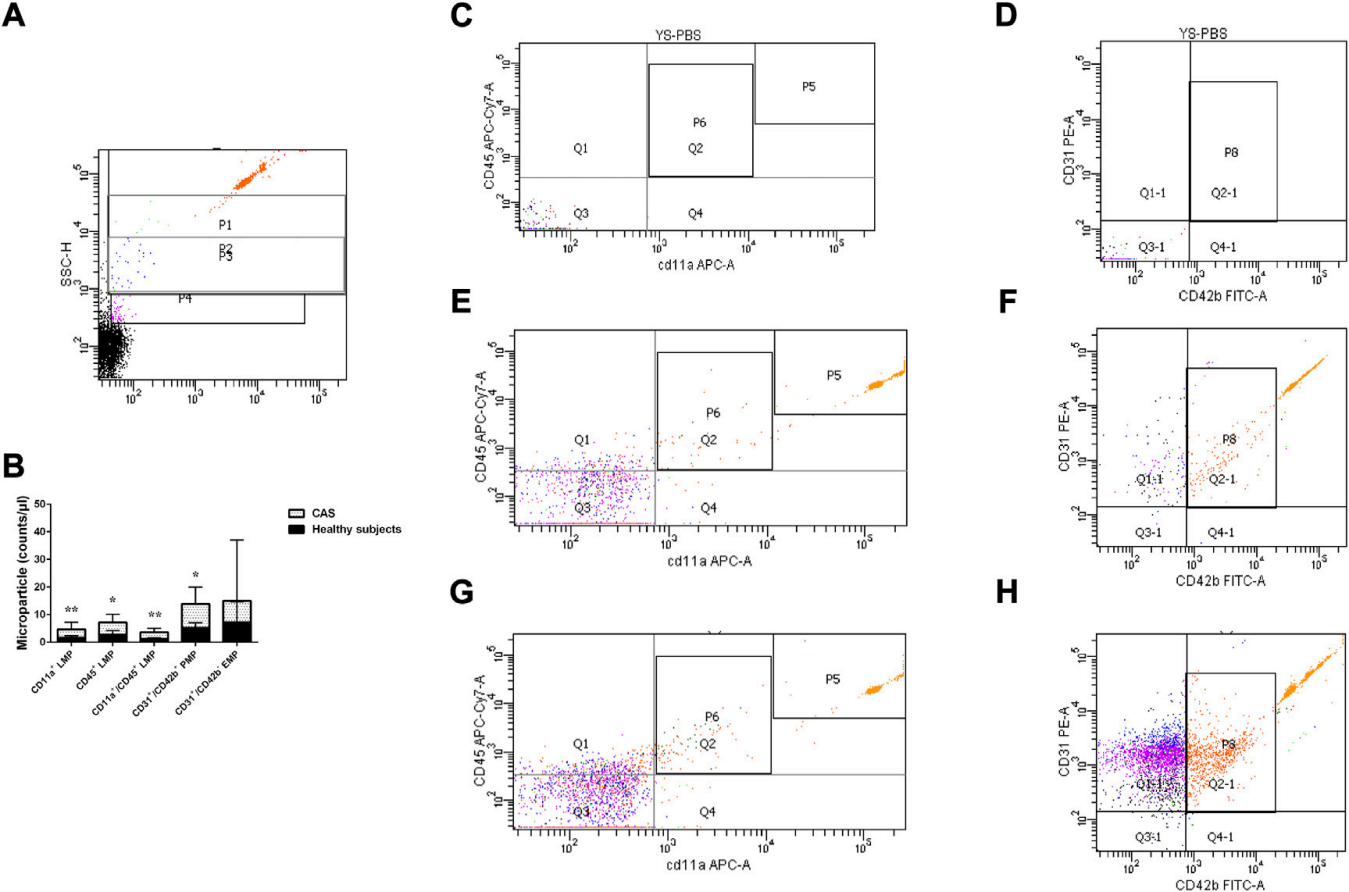
**(A)** P1-P3 represent gates set with bead standards 1, 0.5, and 0.24 μm, respectively. **(B)**. The quantitative expression of LMP, PMP, and EMP in the two groups. **(C,D)** stand for the negative control. **(E,F)** stand for the healthy subjects group, and **(G,H)** stand for the CAS group; P6 represents CD11a^+^/CD45^+^ LMP, Q1&P6 represents CD45^+^ LMP, Q4&P6 represents CD11a^+^ LMP, P8 represents CD31^+^/CD42b^+^ PMP and Q1-1 represents CD31^+^/CD42b^−^ EMP. P5 represents the quantity of micro bead standard. Values are shown as median [25th–75th percentile]. *p < 0.05, **p < 0.01 vs. healthy subjects.

The plasma levels of CD45^+^ LMP, CD11a^+^ LMP, CD11a^+^/CD45^+^ LMP and CD31^+^/CD42b^+^ PMP were significantly higher in CAS patients than healthy subjects (*p* = 0.030, *p* = 0.004, *p* = 0.005, and *p* = 0.020, respectively). CD31^+^/CD42b^−^ EMP showed no difference between CAS patients and healthy subjects (*p* > 0.05) (Figure 2). To explore whether differences in age could have an effect on MPs, we performed a correlation test between the two. Correlations were evaluated using the Spearman rank test. The results showed that there was no correlation between differences in age and the levels of MPs(*r* = 0.023 *p* = 0.879).

### Correlation between five types of microparticles and carotid intima-media thickness, maximal plaque area, and the value of leukocyte-derived microparticle, platelet-derived microparticle as new makers to predict carotid atherosclerosis

**FIGURE 3:**
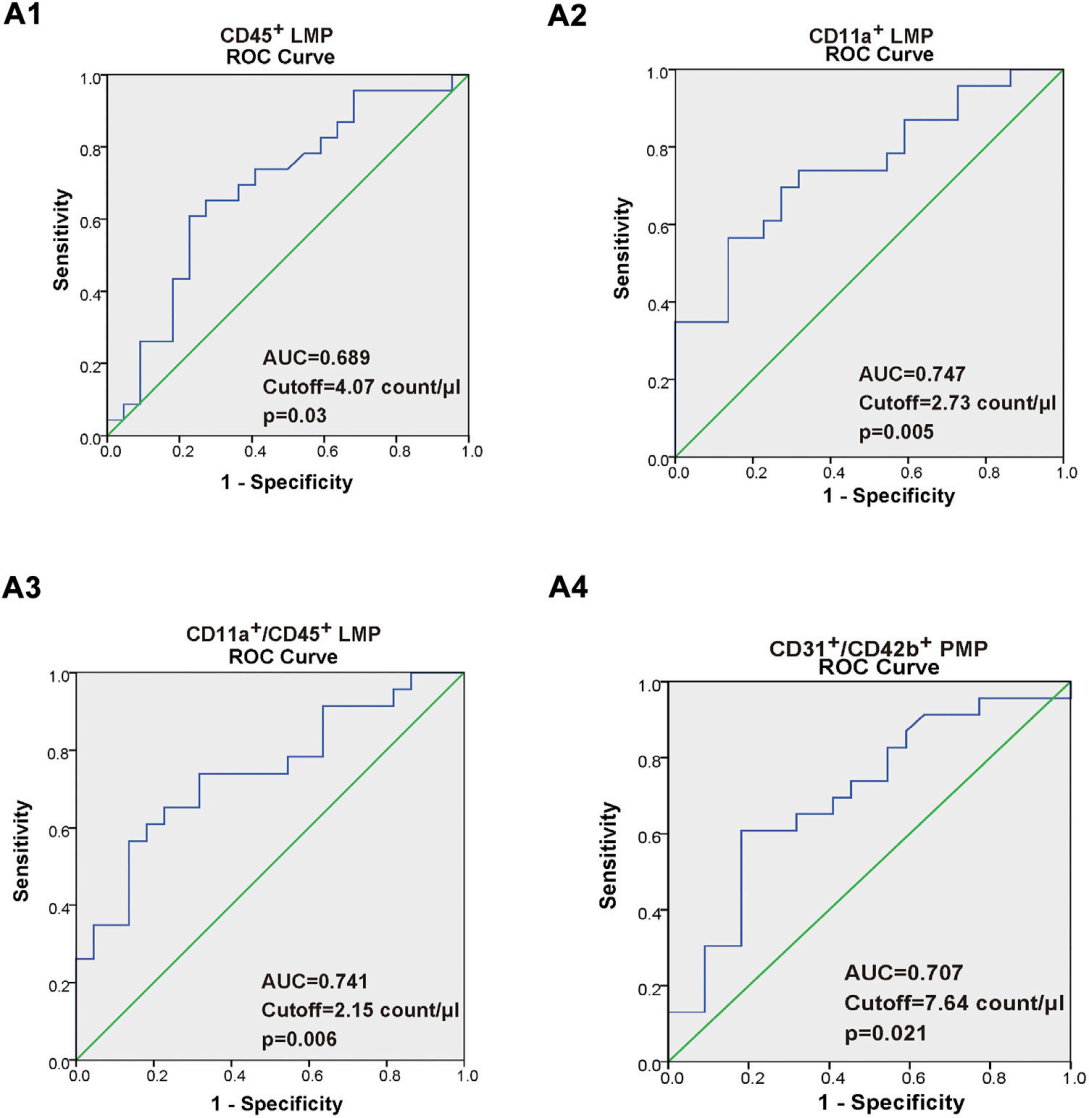
**(A1–A4)** Receiver operating characteristic (ROC) curve of the CD45^+^ LMP, CD11a^+^ LMP, CD11a^+^/CD45^+^ LMP, and CD31^+^/CD42b^+^ PMP. The value of area under the ROC curve (AUC), with a 95% confidence interval for diagnostic experiments, ranged within 0.5–1. AUC = of 0.5 indicates a completely worthless diagnosis, 0.5 < AUC <0.7 indicates a lower diagnostic accuracy, 0.7 < AUC < 0.9 indicates a moderate diagnostic accuracy, 0.9 < AUC < 1, indicates a higher diagnostic accuracy, and AUC = of 1, indicates a completely ideal diagnosis.

As shown in Table 2, we found that in CAS patients, the increment in CD45^+^ LMP, CD11a^+^ LMP, CD11a^+^/CD45^+^ LMP, and CD31^+^/CD42b^+^ PMP were positively related with increasing maximal plaque area. Moreover, the increment in CD11a^+^ LMP and CD11a^+^/CD45^+^ LMP were also positively related with increasing CIMT. Additionally, there were no correlations between CD31^+^/CD42b^+^ PMP, CD31^+^/CD42b^−^ EMP, and CIMT (*p* > 0.05). A ROC curve was constructed to evaluate the ability of CD11a^+^ LMP, CD45^+^ LMP, CD11a^+^/CD45^+^ LMP, and CD31^+^/CD42b^+^ PMP as potential makers to predict CAS, as shown in Figure 3. The area under curve (AUC) of the ROC of the above MPs was 0.689,0.747, 0.741, and 0.701, respectively. AUC values from 0.7 to 0.9 for a diagnostic test represented moderate accuracy.

**TABLE 2:** Correlation analysis of five microparticles with maximal plaque area and CIMT.

	Maximal plaque area		CIMT	
CD45^+^ LMP	*p* = 0.005	*r* = 0.647	*p* = 0.089	*r* = 0.425
CD11a^+^ LMP	*p* = 0.003	*r* = 0.674	*p* = 0.002	*r* = 0.686
CD45^+^/CD11a^+^ LMP	*p* = 0.005	*r* = 0.645	*p* = 0.005	*r* = 0.649
CD31^+^/CD42b^+^PMP	*p* = 0.011	*r* = 0.598	*p* = 0.354	*r* = 0.163
CD31^+^/CD42b^−^ EMP	*p* = 0.211	*r* = 0.416	*p* = 0.206	*r* = 0.427

Spearman rank-correlation test was used to analyze the correlation between two variables; *r* = 0 meant no correlation, |r| < 0.3 meant weak correlation, |r| = 0.3–0.5 meant low correlation, |r| = 0.5–0.8 meant significant correlation, |r| > 0.8 meant highly correlated, and |r| = 1 meant completely correlated.

### The correlation between endothelial nitric oxide synthase and blood lipid level in the carotid atherosclerosis group

**FIGURE 4:**
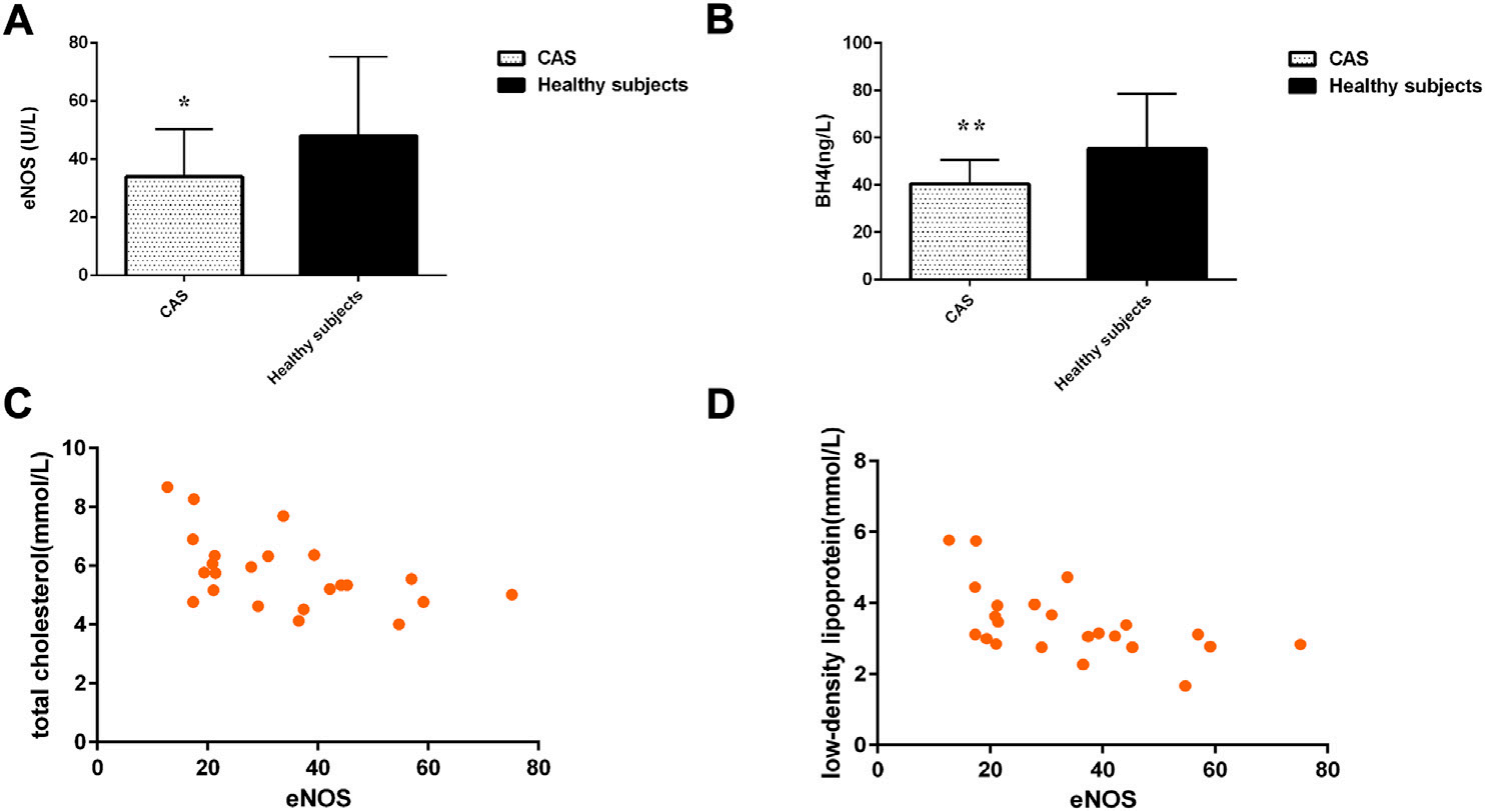
**(A)** The level of eNOS; **(B)** The level of BH4; **(C,D)** Scatter plot of the correlation between the level of eNOS and blood lipid in the CAS group. Values are shown as mean ± SD. *p < 0.05, **p < 0.01 vs. healthy subjects.

The level of eNOS and BH4 in the CAS group was significantly lower than that in healthy subjects (*p* < 0.05) (Figures 4A,B). Spearman rank-correlation test was used to examine the correlation between eNOS expression levels and total cholesterol, triglyceride, low-density lipoprotein cholesterol, and high-density lipoprotein cholesterol levels in the CAS group. The level of plasma eNOS was significantly negatively correlated with total cholesterol level (*r* = −0.512, *p* = 0.012) and low-density lipoprotein level (*r* = −0.581, *p* = 0.004) in CAS patients (Figures 4C,D).

### Effects of circulating microparticles from carotid atherosclerosis on endothelial nitric oxide synthase uncoupling in the cardiovascular system of mice

**FIGURE 5:**
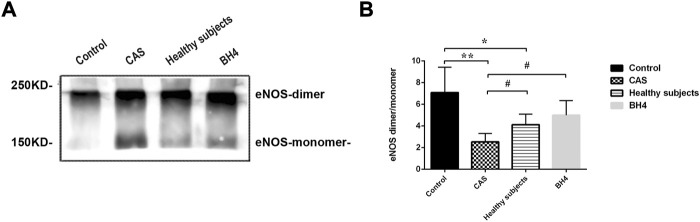
**(A,B)** Detection of eNOS by Western blot (*n* = 4 per group). Values are shown as means ± SD. *p < 0.05, **p < 0.01 vs. control group; ^#^ *p* < 0.05 vs. CAS group.

The lower the ratio of eNOS dimer/eNOS monomer, the more severe the eNOS uncoupling. Twenty-four hours after injecting MPs from CAS patients and healthy subjects, eNOS uncoupling occurred in the circulating tissues of mice. Compared with the control group, the ratio of eNOS dimer/monomer in CAS patients and healthy subjects significantly decreased (*p* = 0.001, *p* = 0.048). However, the degree of uncoupling was more serious in CAS patients than in healthy subjects (*p* = 0.049) and BH4 could inhibit the process in the cardiovascular system caused by circulating MPs in CAS patients (*p* = 0.029) (Figure 5).

### Morphological observation of mice aorta and the expression of vascular cell adhesion molecule-1 in the intima

**FIGURE 6:**
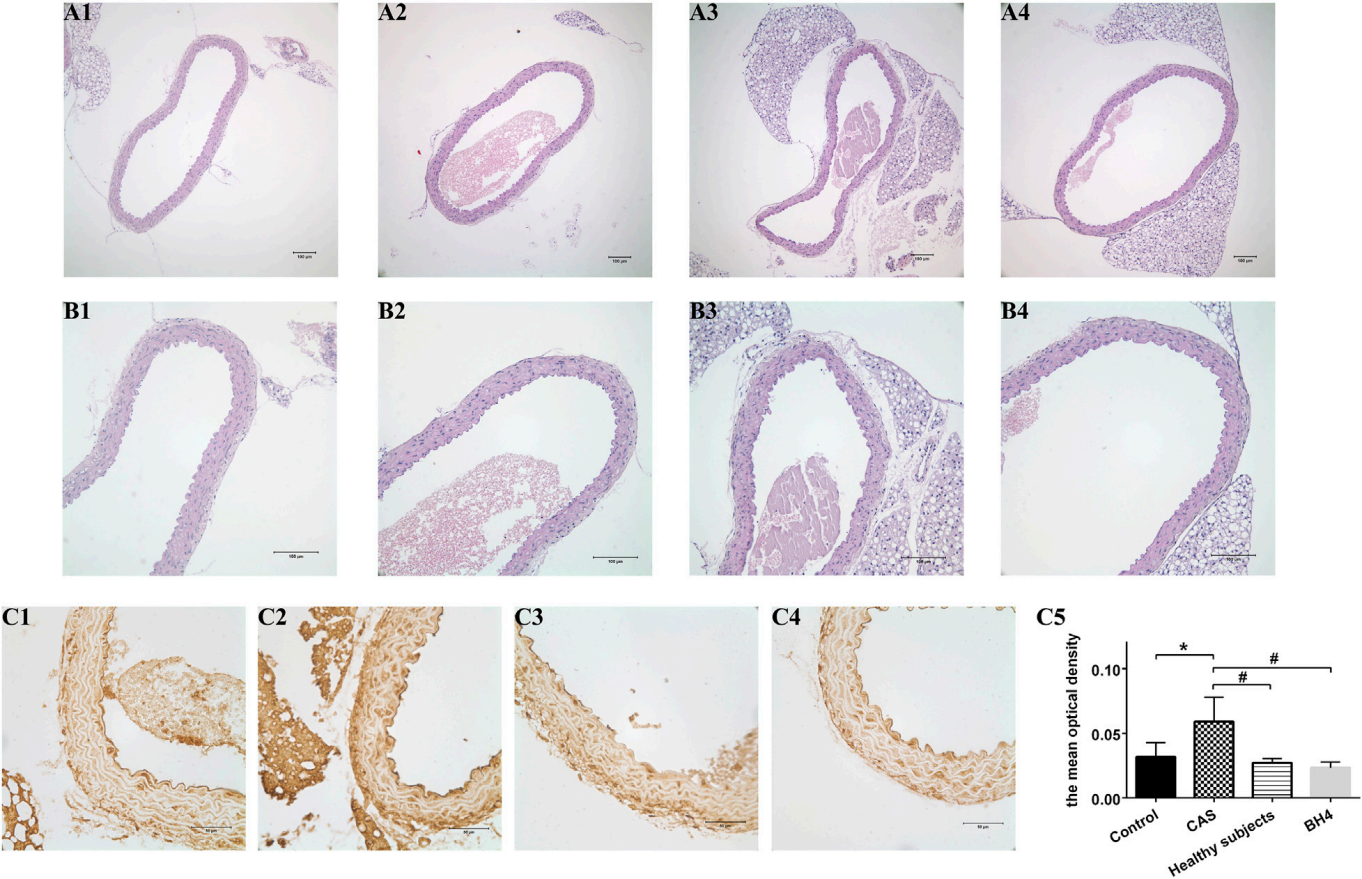
Pathology of mice aorta using HE staining **(A1–B4)** and the expression of VCAM-1 in the intima **(C1–C4)**. **(A1–C1)** The saline control group. **(A2–C2)** The CAS group. **(A3–C3)** The healthy subjects. **(A4–A4)** The BH4 group. **(C5)** The mean optical density of VCAM-1 in the intima of four groups [*n* = 7 per group; **(A1–A4)** ×100, **(B1–B4)** ×400]. Values are shown as means ± SD. *p < 0.05 vs. the control group; ^#^ *p* < 0.05 vs. the CAS group.

The aortic structure of the mice was observed under the microscope. The aortic intima of the four mice groups was intact, smooth, and continuous, and no structural abnormalities were observed in the media and adventitia. The results of immunohistochemistry showed that the level of VCAM-1 in the intimal was significantly increased after the injection of CAS patient MPs compared with the other three groups (Figure 6).

## Discussion

AS is a common pathological basis for various cardiovascular and cerebrovascular events, such as coronary heart disease and stroke (Rautou et al., 2011; Pavlides et al., 2014). Endothelial dysfunction is a key feature of early AS (Schwarz et al., 2018), representing an initial reversible step in the development of atherogenesis (Mudau et al., 2012). Therefore, a search for more potential biomarkers may become an important tool in the prevention or reversal of AS progression. CIMT and plaque in the superficial carotid artery, the artery most susceptible to AS among the systematic arteries, can be clearly observed by color Doppler ultrasonography. Thickening of CIMT is one of the risk factors for cerebral infarction, which has been included in the stroke guidelines by the American Heart Association (Meschia et al., 2014). Recent studies have shown that atherosclerotic plaque area is a powerful predictor of future cardiovascular risk and a sensitive indicator of efficacy evaluation (Inaba et al., 2012). Therefore, CIMT and plaque area reflect the degree and prognosis of AS. However, the predictive power of early AS is relatively poor.

For the past few years, studies have found that the expression of MPs increases significantly in many diseases, such as hypertension, sleep-disordered breathing syndrome, and coronary heart disease, suggesting that MPs might be a potential predictive biological marker (Yun et al., 2010; Cui et al., 2013; Hu et al., 2015). MPs are shed in the form of budding when cells are activated or undergo apoptosis and carry related substances from parental cells, such as RNA, microRNA, and active proteins, and transmit information between cells to mediate changes in cell biological functions and structures (Rautou et al., 2011; Yáñez-Mó et al., 2015). Circulating MPs are derived from multiple cells, e.g., endothelial cells, platelets, leukocytes, monocytes-macrophages, granulocytes (Ridger et al., 2017). Most studies have focused on EMP, PMP, and LMP as factors playing an important role in AS development (Deng et al., 2017; Barbati et al., 2018).

Therefore, we included 23 patients with CAS whose ultrasound testing was unilateral or bilateral CIMT of ≥0.9 mm or carotid plaque formation with local CIMT increased to 1.3 mm but not significant stenosis (≤50%). A total of 22 sex-matched healthy subjects served as control subjects. We tested the level of circulating MPs in CAS patients and healthy subjects, and analyzed the relationship between carotid intima thickness, plaque area, and MPs, to find potential markers for AS prediction. Four fluorescent antibodies were selected for labeling and detection of CD45^+^ LMP, CD11a^+^ LMP, CD11a^+^/CD45^+^ LMP, CD31^+^/CD42b^+^ PMP, and CD31^+^/CD42b^−^ EMP in peripheral blood by flow cytometry. The results showed that the levels of CD45^+^ LMP, CD11a^+^ LMP, CD11a^+^/CD45^+^ LMP, and CD31^+^/CD42b^+^ PMP were significantly elevated in CAS patients. Moreover, we found that the levels of the above-mentioned four MPs were significantly positively correlated with the maximal plaque area. Additionally, the levels of CD11a^+^ LMP and CD11a^+^/CD45^+^ LMP were also significantly positively correlated with CIMT. Based on this linear correlation, we further tested the ability of CD45^+^ LMP, CD11a^+^ LMP, CD11a^+^/CD45^+^ LMP, and CD31^+^/CD42b^+^ PMP as markers to predict CAS development by the ROC curve. The results indicated that CD11a^+^ LMP and CD11a^+^/CD45^+^ LMP represent potential biomarkers for AS prediction and prognosis assessment.

Research on LMP has gradually emerged in recent years, and it is believed that LMP is closely related to AS. They carry markers from their maternal cells including CD45, CD11a, CD11b, CD66b and CD15 that can be detected by flow cytometry using labeled fluorescent antibodies (Anne, 2012). LMPs originating from monocytes, neutrophils and lymphocytes, are involved in the pathological process of AS (Zacharia et al., 2020), promoting endothelial dysfunction, inflammation, thrombosis, matrix degradation, smooth muscle cell phenotypic transformation, cell migration, and angiogenesis (Wang et al., 2013; Loyer et al., 2014; Su et al., 2017). Some previous studies have shown that asymptomatic patients with subclinical AS display increased levels of CD11a LMPs, which are connected with the grade of the intima-media thickness of the carotid artery (Chironi et al., 2010). A recent study from JACC has demonstrated that the level of CD11b/CD66b^+^ LMP is significantly higher in patients with high-grade carotid atherosclerotic stenosis, showing a promising effect on plaque vulnerability (Sarlon-Bartoli et al., 2013). However, the predictive value of LMP in early AS has not been reported. Our results showed that the level of LMP was elevated in CAS patients compared with healthy subjects. At the same time, the ROC curve indicated that CD11a^+^ LMP and CD11a^+^/CD45^+^ LMP had the potential to predict AS occurrence and development.

The role of PMP cannot be ignored. PMP is the most common (70%–90%) isotype of MPs in the blood (Voukalis et al., 2019). Several studies have implied that elevated PMP is associated with endothelial dysfunction and Framingham risk scores for coronary artery disease in healthy subjects (Ueba et al., 2010; Suades et al., 2015). It could promote platelet and fibrin deposition on the arterial wall, directly influencing AS (Suades et al., 2012). The level of CD31^+^/CD42b^+^ PMP was significantly elevated in the peripheral blood of CAS patients in our study, which was in line with the before-mentioned previous report. PMP can also remotely activate leukocytes and platelets, thereby generating more MPs, leaving them in a state of continuous activation and potential disease progression (Montoro-García et al., 2011).

In endothelial cells, the normal structure and function of eNOS is associated with heat-shock protein 90 (Hsp90) (Natarajan et al., 2015; Vellecco et al., 2016). As shown in all eNOS cofactors and chaperone proteins, Hsp90 and BH4 have been proven as determinant converter for eNOS generation of NO or superoxide (Chen et al., 2014; Peng et al., 2015). By binding with eNOS oxygenase domain, Hsp90 keeps eNOS in an active form and inhibits superoxide generation. Inhibition of Hsp90 leads to eNOS uncoupling and superoxide generation (Chen et al., 2014). Previous study showed that MPs derived from acute coronary syndrome (ACS) patients attenuated endothelial-dependent vasodilatation, reduced NO expression and eNOS phosphorylation and decreased the interaction between eNOS and Hsp90, while increasing O_2−_ generation (Han et al., 2015; Wang et al., 2021). However, the association between eNOS/BH4 and MPs is not clear. We found the level of plasma eNOS and BH4 decreased in CAS patients, indicating present endothelial dysfunction in patients. Anti-atherosclerotic NO is physiologically generated by eNOS, while its properties would change after eNOS becomes uncoupled with the deficiency and oxidation of the eNOS cofactor BH4 (Li and Förstermann, 2009; Förstermann and Sessa, 2012). To explore whether the above-mentioned factors are related to MPs, we extracted and isolated circulating MPs from different subjects and injected them into mice. MPs in CAS patients resulted in serious eNOS uncoupling (significantly decreased ratio of eNOS dimer/monomer) in the cardiovascular system of mice. eNOS uncoupling leads to beneficial NO reduction and peroxides increment, causing endothelial dysfunction and AS development. Previous studies have supported our results that the lower levels of the eNOS from the isolated MPs are found in patients with cardiovascular risk factors compared with healthy subjects (Horn et al., 2012). Our study further demonstrated that MPs of CAS patients could cause eNOS uncoupling in the cardiovascular system of normal mice 24 h after the injection. These results suggest that MPs are the injury factor of endothelial dysfunction in early AS.

The main consequence of eNOS uncoupling at the vascular level is endothelial dysfunction. What part might they play in this process? VCAM-1 expressed on vascular endothelial cells can promote the adhesion and migration of monocyte to ECs which represents the initiation of preliminary inflammation in AS. Early expression of VCAM-1 accelerates injury to the vascular endothelium (Preiss and Sattar, 2007). According to the immunohistochemical staining of VCAM-1, the level of VCAM-1 in the intima of mice from MPs of CAS patients was significantly higher than that in the other three groups. It showed that more severe endothelial dysfunction was triggered by enhancing eNOS uncoupling. However, HE staining showed no change in the structure of the artery, meaning that MPs did not affect structure of the artery due to the short injection time.

In conclusion, CD11a^+^ LMP and CD11a^+^/CD45^+^ LMP may be potential biomarkers for predicting CAS in the initial phase. BH4 related eNOS uncoupling occurs in CAS patients, and circulating MPs from them lead to endothelial dysfunction through eNOS uncoupling. MPs are an earlier indicator than changes in the endothelial structure.

## Limitations

Our study had several limitations. Due to the small sample size, the results should be considered primary. Future studies with a larger cohort will be conducive to reducing possible bias. Although our results showed that circulating MPs could induce uncoupling of eNOS, which type of MPs take a major part and the exact molecular mechanism responsible need to be further explored *via in vitro* experiments.

## Data Availability

The original contributions presented in the study are included in the article/supplementary material, further inquiries can be directed to the corresponding author.

## Abbreviations

AS, atherosclerosis; CAS, carotid atherosclerosis; CIMT, carotid intima-media thickness; EMP, endothelial cell-derived microparticle; eNOS, endothelial nitric oxide synthase; LMP, leukocyte-derived microparticle; MP, microparticle; NO, nitric oxide; ROC, receiver operating characteristic; PMP, platelet-derived microparticle; VCAM-1, vascular cell adhesion molecule-1.
